# Optilume Drug-Coated Balloon for Acute Urinary Retention After Failed Treatment for Complex Recurrent Urethral Stricture Disease

**DOI:** 10.3390/medicina61040700

**Published:** 2025-04-11

**Authors:** Lukas Andrius Jelisejevas, Peter Rehder, Jannik Wassermann, Patricia Kink, Gennadi Tulchiner

**Affiliations:** 1 Department of Urology, Medical University Innsbruck, 6020 Innsbruck, Tyrol, Austria; peter.rehder@i-med.ac.at (P.R.); jannik.wassermann@outlook.de (J.W.); patricia.kink@tirol-kliniken.at (P.K.); gennadi.tulchiner@i-med.ac.at (G.T.); 2 Department of Urology, Tauernklinikum Zell am See, 6700 Zell am See, Salzburg, Austria

**Keywords:** urethral stricture, urinary retention, paclitaxel, urinary bladder neck obstruction, urinary catheterisation, urethral catheters, urinary diversion, lower urinary tract symptoms, cystoscopy

## Abstract

*Background and Objectives*: We aimed to assess the outcomes of upfront Optilume drug-coated balloon (DCB) dilation in patients after failed treatment for complex recurrent urethral stricture disease. All patients presented with acute urinary retention and were treated with DCB dilation regardless of stricture site and length. *Materials and Methods*: We retrospectively evaluated patients with acute urinary retention and known complex recurrent urethral strictures. Patients presented at the urology emergency room of our tertiary centre with an inability to void or a post-void residual (PVR) volume exceeding 400 mL between August 2021 and February 2024. Urethrography and/or endoscopic imaging confirmed the diagnosis. Patients with urinary tract infection/sepsis and those with neurological disease were excluded. Urethral dilation to 20 Fr was performed, followed by DCB dilation (30 Fr, 10 bar, 10 min). The primary endpoints were anatomical success (≥14 Fr by cystoscopy/calibration) at 12 months and freedom from repeat interventions. *Results*: Thirty-one consecutive male patients were evaluated, with twenty-six patients followed for ≥12 months (mean age 65 ± 16.8 years). The stricture sites included seven bulbopenile, seven bulbomembranous, seven anastomotic, three bladder neck, one penile, and one panurethral stricture. The median number of prior urethral/surgical interventions was 2 [IQR: 1–3] (range: 1–31). The median stricture length was 3 [IQR: 2–4] cm (range: 1–8). At 12 months, 65.4% (17/26) of subjects voided satisfactorily and were free of recurrence and reoperation. *Conclusions*: Timely DCB dilation may offer a viable treatment option for patients with complex recurrent urethral strictures and urinary retention, particularly those who are unable or unwilling to undergo surgical reconstruction and prefer to avoid indwelling catheters.

## 1. Introduction

In December 2021, the Optilume drug-coated balloon (DCB) (Laborie, Plymouth, MN, USA) received FDA approval as a novel approach for managing recurrent anterior urethral strictures. This device combines coaxial mechanical dilation with the local delivery of the antimitotic agent, paclitaxel, which inhibits cell proliferation. The prospective ROBUST trials demonstrated the safety, efficacy, and superiority of DCB dilation over standard endoscopic treatments for recurrent short bulbar urethral strictures, with follow-up extending up to five years [1,2]. Current evidence supports the use of a DCB for recurrent anterior strictures measuring less than 3 cm. However, its potential role in treating longer strictures, other anatomical sites, and patients with complicating factors—such as prior irradiation, prior urethroplasty, or lichen sclerosus—remains unclear [3,4,5,6]. There is a knowledge gap regarding the use of DCBs for strictures longer than 3 cm and in locations beyond the anterior urethra. Drawing from our own experiences and those of others with DCB dilation, we expanded the treatment indications to include cases beyond isolated anterior strictures. To date, no studies have investigated DCB dilation for the treatment of urethral strictures in the context of acute urinary retention. This study aims to evaluate the outcomes of immediate drug-coated balloon treatment in a real-world cohort of multimorbid patients with complex recurrent urethral strictures, presenting with acute urinary retention, irrespective of stricture length or location.

## 2. Methods

### 2.1. Study Population

**Figure 1:**
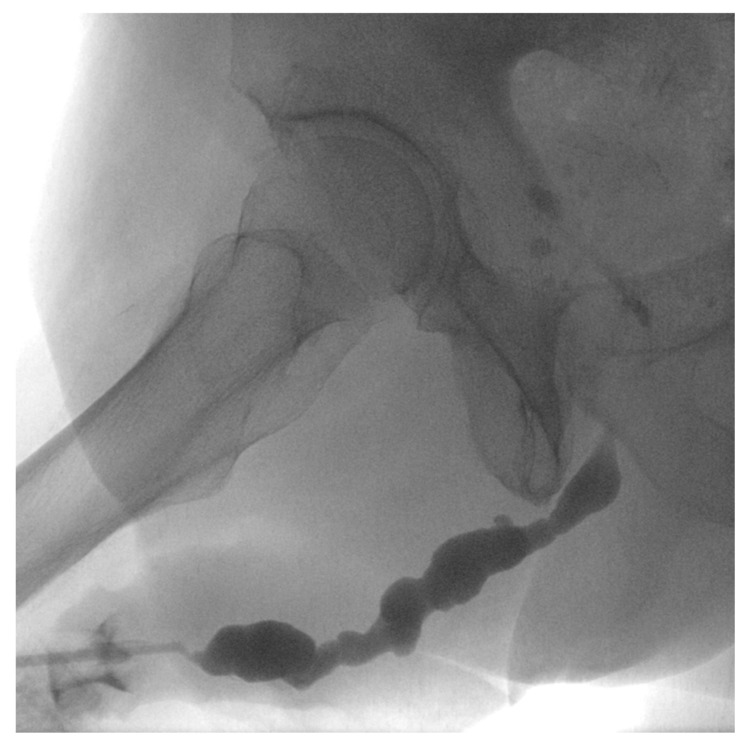
Retrograde urethrography showing multiple urethral strictures after previous urethroplasty.

**Figure 2:**
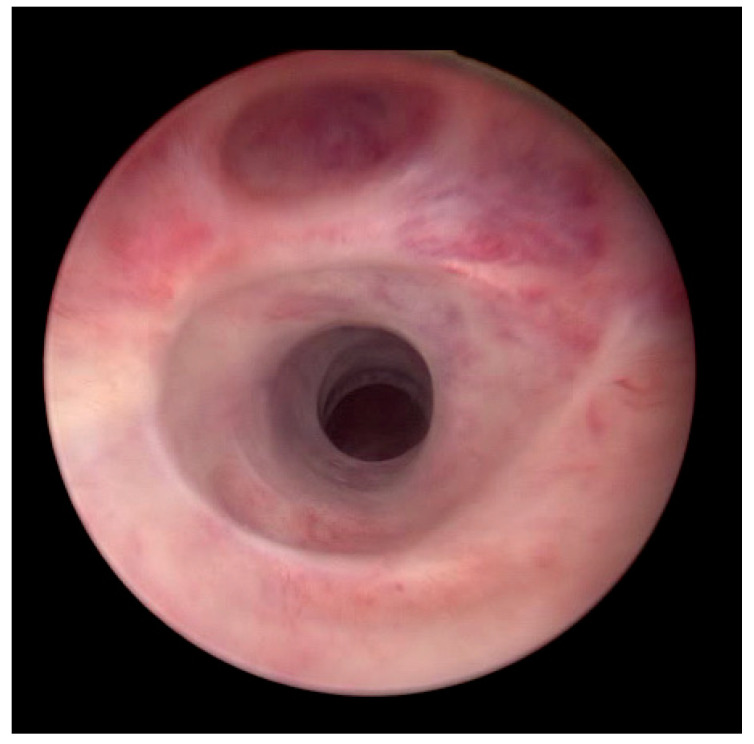
Endoscopic view of a complex bulbar stricture.

We performed a retrospective analysis of patients who underwent upfront DCB treatment for urethral stricture disease at a single tertiary care centre. During the study period, 134 patients underwent DCB dilation and were prospectively followed according to our study protocol. We included male patients aged ≥18 years with previously treated complex urethral stricture disease, regardless of location and length, who presented at our urology emergency department with urinary retention. We defined a complex urethral stricture as a stricture longer than 3 cm or one that has failed at least three prior treatments, including endoscopic and open surgical reconstruction (see Figure 1 and Figure 2). The diagnosis of urethral stricture disease was based on the inability to pass a urethral catheter, retrograde urethrography, or careful endoscopy. Urinary retention was defined as the inability to pass urine and/or post-void residual volume >400 mL. We excluded patients with urinary tract infection or neurological disease. Written informed consent was obtained prior to treatment. All patients preferred an endoscopic approach to open surgery. The institutional review board approved this study (study number: 1101/2022).

### 2.2. Surgical Procedure and Follow-Up

**Figure 3:**
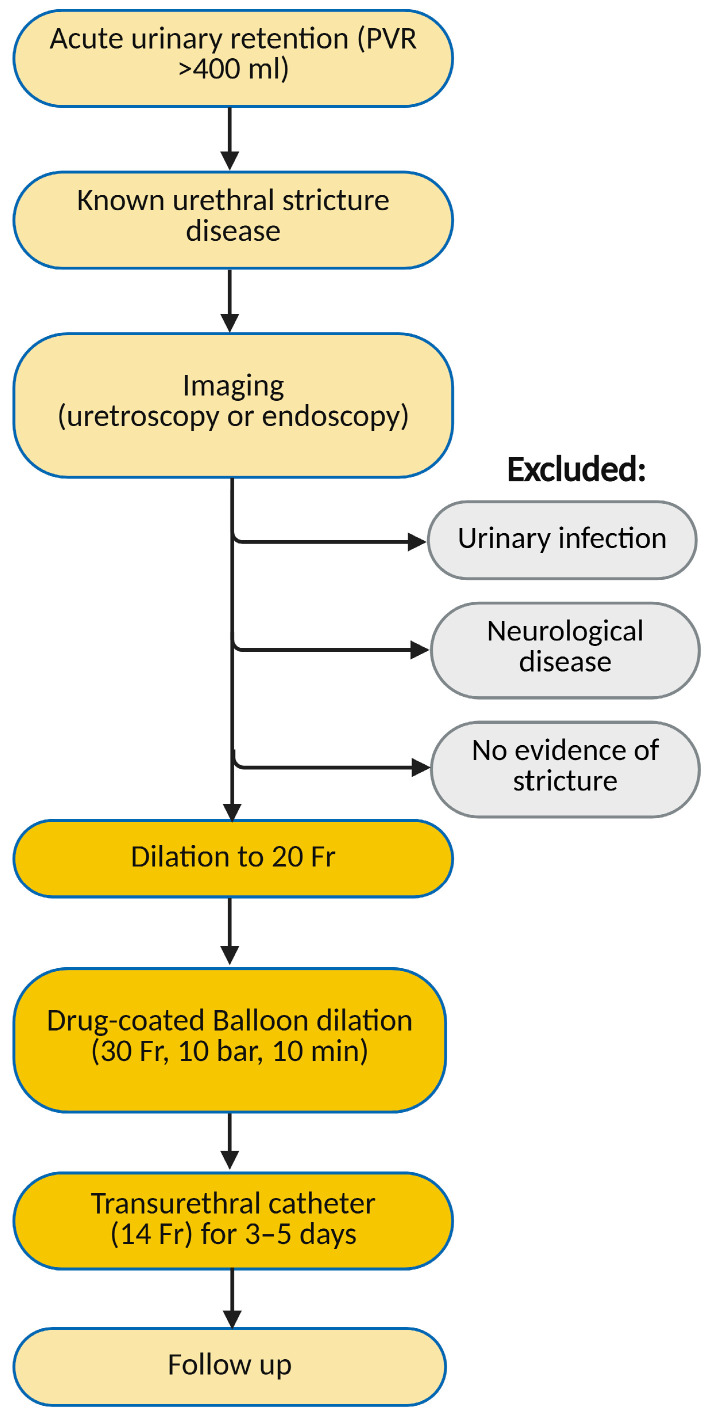
Flowchart depicting inclusion and exclusion criteria.

The patient was placed in the lithotomy position. The procedure was performed under either local or general anaesthesia, depending on patient preference. For local anaesthesia, 22 mL of intraurethral 2% lidocaine hydrochloride gel was used. No premedication was used. Urethrocystoscopy was performed to evaluate the stricture and exclude other pathologies. A 0.035″ hydrophilic guidewire was then passed under cystoscopic guidance. The urethra at the stricture site was pre-dilated to 20 Fr using hydrophilic urethral dilators. The inflation device was filled with 20 mL of sterile saline. The DCB was positioned over the guidewire at the stricture site. A 5 cm, 30 Fr DCB was used to ensure at least 0.5 cm of overlap proximally and distally to the stricture. For longer strictures, two balloons were used sequentially, starting with proximal and followed by distal dilation. The balloon was inflated to the recommended pressure of 10 bar, which was maintained for 10 min before deflation and removal. A 14 Fr Foley catheter was inserted for 3 to 5 days. We advised patients to use barrier contraception for 30 days after treatment to avoid exposure of sexual partners to paclitaxel. Men with partners of child-bearing potential were advised to use barrier contraception for at least 12 months. Follow-up appointments were conducted at 30 days and then every 3 months thereafter (see Figure 3).

### 2.3. Study Endpoint and Statistical Analysis

The primary study endpoints were anatomical success (defined as ≥14 Fr by cystoscopy or calibration) at 12 months, along with freedom from reintervention. We defined reintervention as any treatment for urethral strictures, subsequent to the DCB procedure. Statistical analysis was conducted using SPSS Version 28. Categorical variables were compared using the chi-squared test. Quantitative variables were summarised as mean ± standard deviation or median [interquartile range, IQR] depending on their distribution, which was analysed using the Kolmogorov–Smirnov test. Mann–Whitney U and Student’s *t*-tests were utilised to compare quantitative variables, as appropriate. Stricture length, location, aetiology, number of prior treatments, and radiation history were considered as variables in the statistical analysis.

## 3. Results

Between August 2021 and February 2024, 31 patients with complex recurrent urethral strictures presented with urinary retention and underwent upfront Optilume DCB treatment. Patient characteristics are summarised in Table 1. Prior interventions included simple urethral dilation (including self-dilation), DVIU with or without intralesional mitomycin C, open urethroplasty, bladder neck incision, and anastomotic incision. Five recurrence-free patients had not completed 12 months of follow-up at the time of data analysis and were therefore excluded from subsequent analyses.

### Outcomes at 12-Month Follow-Up

**Figure 4:**
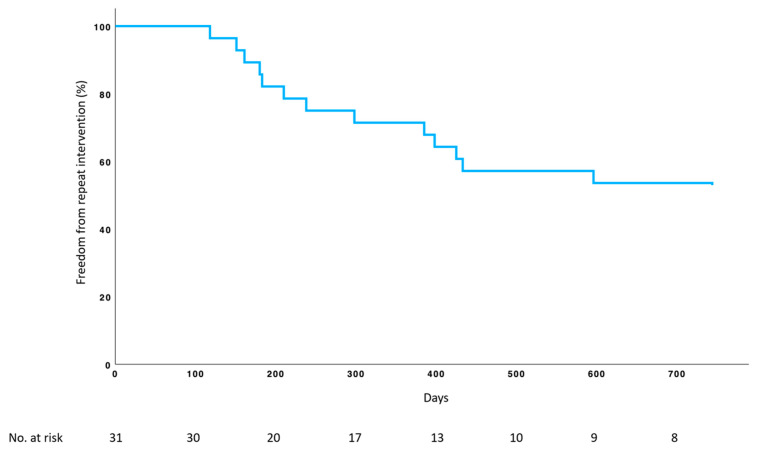
Kaplan–Meier curve for freedom from repeat intervention.

In the 26 patients with a minimum follow-up of 12 months, 17 (65.4%) were free from recurrence and repeat interventions, voided satisfactorily, and did not require any form of catheterisation (see Figure 4). Considering the anatomical site, freedom from recurrence was 29% (2 out of 7) for anastomotic, 71% (5 out of 7) for bulbomembranous, 71% (5 out of 7) for penobulbar, 100% (3 out of 3) for bladder neck and 100% (1 out of 1) for penile strictures. One patient with panurethral stricture (8 cm) remained recurrence-free for 13 months. Eight patients experienced sustained freedom from reintervention after 24 months. In descriptive analysis, patients without stricture recurrence had a higher number of prior treatments (3.5 [IQR 2–10.5] vs. 2 [IQR 1–2.5] pre-treatments, *p* = 0.02) and longer strictures (3.75 [IQR 3–5.75] vs. 2.5 [IQR 2–3] cm, *p* = 0.03). Prior irradiation was associated with stricture recurrence (*p* = 0.01). Both groups did not differ with regard to age, body mass index, diabetes, coronary heart disease, and smoking. The mean PVR volume was significantly improved to 21.1 ± 28.0 mL. No serious adverse events related to DCB treatment were observed. Adverse events such as dysuria (n = 3, 9.7%, Clavien–Dindo Grade 1) and urethral bleeding (n = 1, 3.2%, Clavien–Dindo Grade 1) resolved within the first 7 days. Although approximately half of the strictures involved the membranous urethra and/or the vesicourethral anastomosis, urinary continence was not compromised. There was no change in the original continence after the 30 Fr dilation of the membranous/sphincteric urethra. During the follow-up period, one patient underwent cystectomy with ileal conduit diversion due to bladder cancer at 16 months. Two patients opted for definitive surgical treatment and underwent successful non-transecting anastomotic bulbar urethroplasty at 10 and 15 months.

## 4. Discussion

### 4.1. Background and Clinical Guidelines

Urethral stricture disease is relatively common, affecting an estimated 229 to 627 males per 100,000 individuals [7]. Endoscopic treatments such as urethral dilation and direct vision internal urethrotomy (DVIU) are usually utilised as first-line interventions. Recurrence is common, with most cases reappearing within 12 months [8,9]. Current guidelines recommend urethroplasty as the standard intervention for recurrent cases [10,11]. Recurrence rates following urethroplasty may be underreported [12]. Redo urethroplasty poses significant challenges, and currently, no universally accepted guidelines exist for managing these complex cases [13].

DCB dilation was recently incorporated into the European Association of Urology (EAU) guidelines, which provide a weak recommendation for its use in short (<3 cm) bulbar strictures with at least two prior endoscopic treatments, but only in patients for whom urethroplasty is not an option [14]. The American Urological Association (AUA) guidelines offer a conditional (Grade B) recommendation for DCB in combination with urethral dilation or DVIU, allowing its use for recurrent bulbar urethral strictures <3 cm in length, regardless of previous treatments [11].

### 4.2. Current Evidence

The prospective first-in-man ROBUST I study evaluated the outcomes of DCB dilation in 53 patients with short (≤2 cm) recurrent bulbar urethral strictures, providing the longest follow-up to date. It demonstrated a functional success rate of 58% and an estimated freedom from repeat intervention of 71.7% at 5 years. There were no serious treatment-related adverse events, and erectile function was not affected. Treatment success was not dependent on stricture characteristics (stricture length, number of prior treatments), but correlated strongly with balloon size. Functional success, improvement of Qmax and PVR were significantly better when using a 30 Fr DCB as compared to 24 Fr [2]. These findings align with our experience, and we now exclusively use the 30 Fr DCB.

The ROBUST III trial (prospective, multicentre, single blind, randomised controlled study) randomised 127 participants 2:1 to receive treatment with the Optilume DCB or endoscopic management (standard of care) [1]. Most patients treated with DCBs had short (average length 1.63 cm) bulbar (89.9%) strictures. It showed maintained improvement in both objective and subjective voiding parameters over a 2-year follow-up.

Several additional studies, beyond the approval trials, have reported the results of DCB treatment. A multicentre study conducted in Spain reported a treatment success rate of 73.8% in patients predominantly with bulbar strictures (median stricture length 1.5 cm (0.5–5.3)), with a median follow-up of 8 months [15]. In another study by Alhamdani et al., a small real-world cohort of 17 patients exhibited a success rate of 76% at the 30-month follow-up [4]. Additionally, another study group demonstrated a 90.7% rate of freedom from reintervention among 43 patients during a follow-up period of at least 7 months [16]. Optilume DCB treatment following failed urethroplasty achieves success rates comparable to those observed in patients without prior urethral reconstruction, as evidenced by short-term follow-up at 3 months [17]. These studies primarily focused on short bulbar strictures and had limited follow-up durations.

### 4.3. Study Rationale and Patient Selection

This study specifically evaluated a select subgroup of patients with a history of prior stricture treatments who presented with acute urinary retention as an emergency. Many of these individuals were unsuitable for surgery due to comorbidities, while others were discouraged by previous treatment experiences and reluctant to undergo further invasive interventions. Additionally, some patients hesitated to pursue reconstructive surgery due to concerns about potential complications, such as erectile dysfunction and altered glans sensitivity. Delays in definitive reconstruction are also common. A study by Hoy et al. reported a median waiting time of 151 days, during which 15.9% of patients experienced complications [18]. Given these challenges, combining urethral predilation with DCB dilation was a logical approach to optimise limited hospital resources and provide timely treatment for this high-risk patient population.

### 4.4. Clinical Implications of Our Findings

In our real-world cohort of patients with complex, previously treated recurrent urethral stricture disease who presented with acute urinary retention, DCB dilation resulted in a 65.4% (n = 26) success rate at a 12-month follow-up. Given the challenging patient characteristics—long strictures, various anatomical sites, prior interventions including failed urethroplasty, history of irradiation, and multimorbidity—these outcomes are both surprising and encouraging. In our experience, multimorbid patients typically prefer less invasive therapies, such as DCB dilation in this instance, particularly when the procedure can be performed under local anaesthesia in an outpatient setting. It is noteworthy that patients with more prior treatments and longer strictures achieved better outcomes than those with fewer previous interventions. A possible explanation is that earlier treatments—such as simple dilation, direct vision internal urethrotomy (DVIU ± mitomycin C), or urethroplasty—may have optimised the urethra for DCB dilation. In particular, previous urethroplasty may have contributed to this improvement by reducing excess scar tissue.

### 4.5. Study Limitations

Several limitations impact the interpretation of our findings, including the small sample size, variability in stricture anatomy and prior treatments, and the absence of a control group. Additionally, the single-centre design may limit the generalisability of these results to broader populations. We also acknowledge the lack of standardised questionnaires, which were not employed due to the acute nature of the study setting. Furthermore, obtaining reliable uroflowmetry measurements was challenging, leading to its exclusion from the analysis. The use of at-home uroflowmetry could offer an alternative for future studies [19]. To establish evidence-based recommendations, larger prospective randomised trials are needed.

## 5. Conclusions

Timely DCB dilation may offer a viable treatment option for patients with complex recurrent urethral strictures and urinary retention, particularly those who are unable or unwilling to undergo surgical reconstruction and prefer to avoid indwelling catheters.

## Figures and Tables

**Table 1:** Descriptive characteristics for 31 patients treated with Optilume DCB.

Parameter		Result
Age (years)		65 ± 15.4 (range: 22–83)
Body mass index (kg/m^2^)		27.5 ± 4.99 (range: 18.7–38.1)
Prior interventions		2 [IQR: 1–3] (range: 1–31)
PVR (baseline) (mL), mean ± SD		680.3 ± 352.3 mL
PVR (at 12 months) (mL)		21.1 ± 28.0 mL
Follow-up (months)		13.3 ± 11.4 (4–40)
Smoker		
	Yes	5 (16.1%)
	No	26 (83.9%)
Diabetes		
	Yes	6 (19.4%)
	No	25 (80.6%)
Irradiation		
	Yes	6 (19.4%)
	No	25 (80.6%)
Aetiology		
	Instrumentation	16 (51.6%)
	Prostatectomy	7 (22.6%)
	Idiopathic	3 (9.7%)
	Prior hypospadias repair	3 (9.7%)
	Lichen sclerosus	2 (6.5%)
Stricture location		
	Anastomotic	8 (25.8%)
	Penobulbar	7 (22.6%)
	Bladder neck	6 (19.4%)
	Bulbomembranous	7 (22.6%)
	Penile	2 (6.5%)
	Panurethral	1 (3.2%)
Stricture length (cm)		3 [IQR: 2–4] (range: 1–8)
	<2 cm	7 (22.6%)
	2–3 cm	14 (45.1%)
	>3 cm	10 (32.3%)
Complications (Clavien–Dindo)		
	None	27 (87%)
	Grade 1	4 (12.9%)

## Data Availability

The original contributions presented in this study are included in the article. Further inquiries can be directed to the corresponding author.

## References

[1] VanDyke M.E., Morey A.F., Coutinho K., Robertson K.J., D’Anna R., Chevli K., Cantrill C.H., Ehlert M.J., Te A.E., Dann J. (2024). Optilume drug-coated balloon for anterior urethral stricture: 2-year results of the ROBUST III trial. BJUI Compass.

[2] DeLong J., Virasoro R., Pichardo M., Estrella R., Lay R.R., Espino G., Elliott S. (2024). Long-Term Outcomes of Recurrent Bulbar Urethral Stricture Treatment with the Optilume Drug-Coated Balloon: Five-Year Results from the ROBUST I Study. J. Urol..

[3] Sitharthan D., Islam S., Razi B., Dhar A., Vass J. (2024). Optilume^®^ drug-coated balloon dilation for the treatment of refractory post-TURP bladder neck contracture. Urol. Case Rep..

[4] Alhamdani Z., Ong S., Zhong W., Chin P. (2024). Laborie Optilume^®^ Drug-coated balloon may lower the re-treatment rate post-intervention for challenging urethral stricture disease in long-term follow-up: A prospective cohort study. J. Endourol..

[5] Stuehmeier J., Jelisejevas L.A., Kink P., Gulacsi A., Horninger W., Rehder P. (2021). Optilume^®^ drug-coated balloon dilation in complex female urethral stricture. Urol. Case Rep..

[6] Estaphanous P., Khalifa A.O., Makar Y. (2024). Efficacy and Safety of Optilume Drug-Coated Balloon for Urethral Stricture Treatment: A Systematic Review and Meta-Analysis. Cureus.

[7] Rourke K., Hickle J. (2012). The clinical spectrum of the presenting signs and symptoms of anterior urethral stricture: Detailed analysis of a single institutional cohort. Urology.

[8] Barbagli G., Fossati N., Montorsi F., Balò S., Rimondi C., Larcher A., Sansalone S., Butnaru D., Lazzeri M. (2020). Focus on Internal Urethrotomy as Primary Treatment for Untreated Bulbar Urethral Strictures: Results from a Multivariable Analysis. Eur. Urol. Focus.

[9] Steenkamp J.W., Heyns C.F., de Kock M.L. (1997). Internal urethrotomy versus dilation as treatment for male urethral strictures: A prospective, randomized comparison. J. Urol..

[10] Lumen N., Campos-Juanatey F., Greenwell T. (2021). European Association of Urology Guidelines on Urethral Stricture Disease (Part 1): Management of Male Urethral Stricture Disease. Eur. Urol..

[11] Wessells H., Morey A., Souter L., Rahimi L., Vanni A. (2023). Urethral Stricture Disease Guideline Amendment (2023). J. Urol..

[12] Jasionowska S., Brunckhorst O., Rees R.W., Muneer A., Ahmed K. (2019). Redo-urethroplasty for the management of recurrent urethral strictures in males: A systematic review. World J. Urol..

[13] Verla W., Waterloos M., Spinoit A.-F., Buelens S., De Bleser E., Oosterlinck W., Martins F., Palminteri E., Ploumidis A., Lumen N. (2020). Primary versus Redo Urethroplasty: Results from a Single-Center Comparative Analysis. BioMed Res. Int..

[14] Lumen N., Campos-Juanatey F., Dimitropoulos K., Greenwell T., Martins F.E., Osman N.I., Riechardt S., Waterloos M., Barratt R., Chan G. EAU Guidelines on urethral strictures. Proceedings of the EAU Annual Congress.

[15] Ballesteros Ruiz C., Campos-Juanatey F., Povo Martín I., Biosca S.M., Cardesa Ó.G., Guevara J.A., Formoso N.G., Pascual E.F., Salamanca J.M., Pérez S.M. (2024). Efficacy and safety of Optilume^®^ paclitaxel-coated urethral dilatation balloon in real-life: Experience in a Spanish multicenter study. Actas Urol. Esp..

[16] Mahenthiran A.K., Burns R.T., Soyster M.E., Black M., Arnold P.J., Love H.L., Mellon M.J. (2024). A single-institution experience with the Optilume Urethral Drug Coated Balloon for management of urethral stricture disease. Transl. Androl. Urol..

[17] VVanDyke M., Joshi E., Ceballos B., Baumgarten A., Matz E., Graham K.S., McKibben M.J., Imam A., Wiegand L., Franzen B. (2025). Efficacy of the Optilume paclitaxel drug-coated balloon after urethroplasty: Short-term results from a multicenter study. Ther. Adv. Urol..

[18] Hoy N.Y., Chapman D.W., Dean N., Rourke K.F. (2018). Incidence and Predictors of Complications due to Urethral Stricture in Patients Awaiting Urethroplasty. J. Urol..

[19] Pandolfo S.D., Crauso F., Aveta A., Cilio S., Barone B., Napolitano L., Scarpato A., Mirto B.F., Serino F., Del Giudice F. (2023). A Novel Low-Cost Uroflowmetry for Patient Telemonitoring. Int. J. Env. Res. Public Health.

